# Supplementary material for: MglA functions as a three-state GTPase to control movement reversals of Myxococcus xanthus
Source: Nat Commun. 2019 Nov 22;10:5300. doi: 10.1038/s41467-019-13274-3 (PMC6876712; doi:10.1038/s41467-019-13274-3)
Supplement: Supplementary file 1 — Supplementary Information [file 41467_2019_13274_MOESM1_ESM.pdf]

MglA functions as a three-state GTPase to control movement reversals of Myxococcus xanthus

Galicia et al.

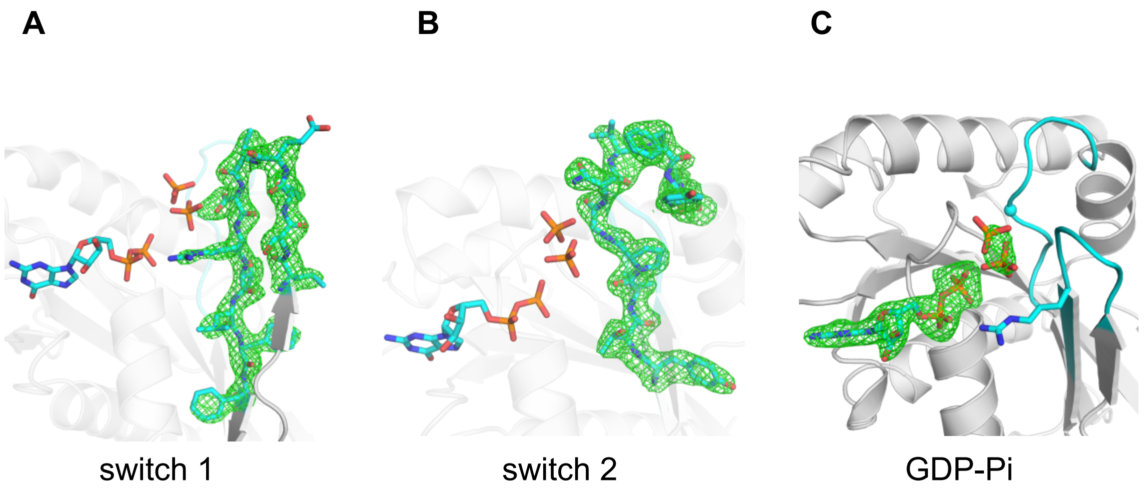

Supplementary Figure 1: Crystallographic analysis of the mixed MglA form.

Omit maps (in green) of the switch 1 (**A**), switch 2 (**B**) and GDP-Pi (**C**). Omit maps were calculated by deleting the region from the model and running a single cycle of refinement in phenix.refine. The calculated Fo-Fc map was then plotted in Pymol at a sigma= 3.0. The refined model is superposed, showing the excellent fit.

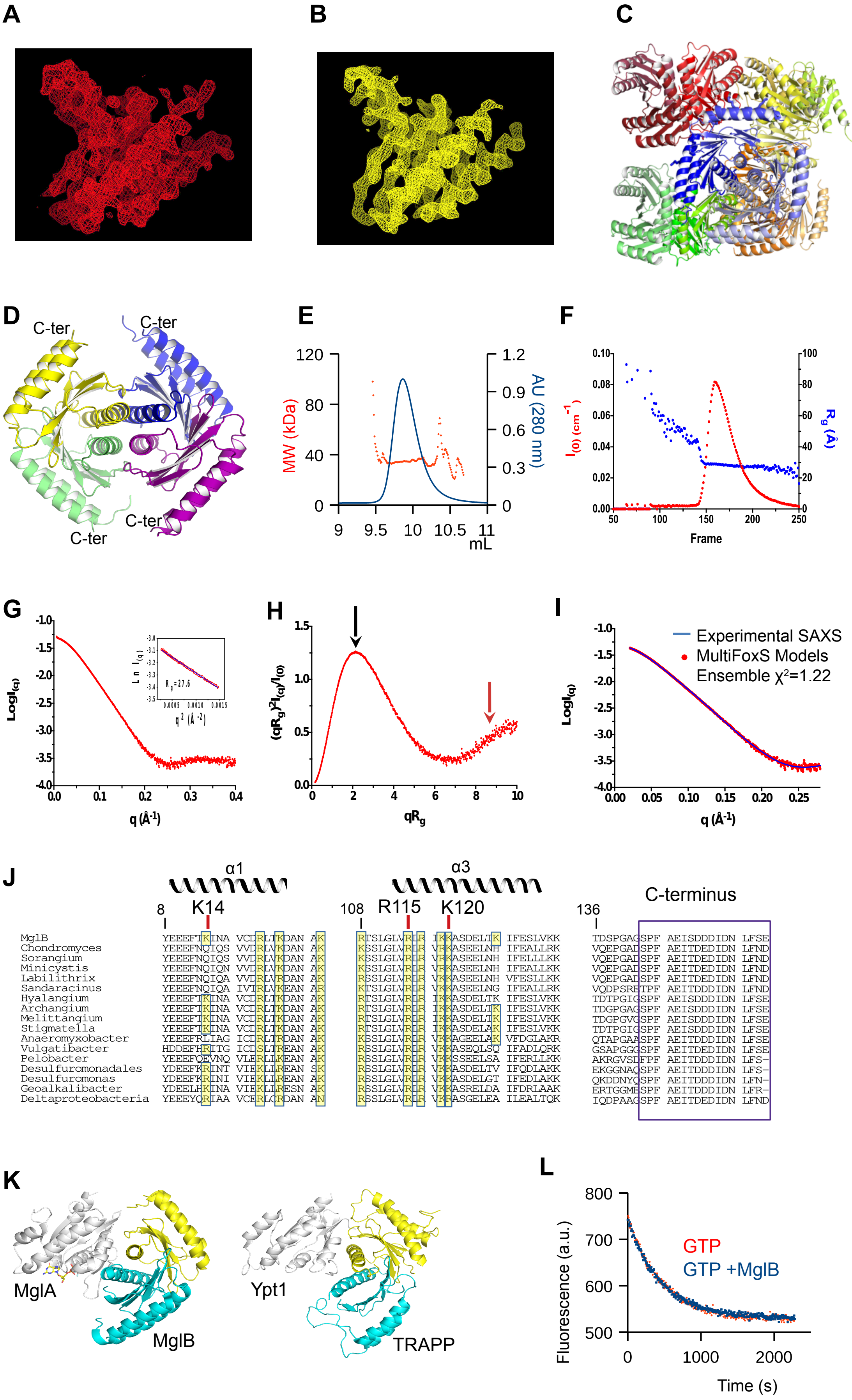

A
B
C
D
C-ter
C-ter
C-ter
C-ter
E
MW (kDa)
AU (280 nm)
mL
F
I(0) (cm-1)
Rg (Å)
Frame
G
LogI(q)
q (Å-1)
Ln I(q)
Rg=27.6
q2 (Å-2)
H
(qRg)2I(q)/I(0)
qRg
I
Experimental SAXS
MultiFoxS Models
Ensemble χ2=1.22
LogI(q)
q (Å-1)
J
α1
α3
K14
R115
K120
C-terminus
MglB
Chondromyces
Sorangium
Minicystis
Labilithrix
Sandaracinus
Hyalangium
Archangium
Melittangium
Stigmatella
Anaeromyxobacter
Vulgatibacter
Pelobacter
Desulfuromonadales
Desulfuromonas
Geoalkalibacter
Deltaproteobacteria
K
MglA
MglB
Ypt1
TRAPP
L
Fluorescence (a.u.)
GTP
GTP +MglB
Time (s)

Supplementary Figure 2. **Crystallographic and solution structural analysis of MglB.**

A- 4-fold NCS-averaged electron density of one MglB monomer in crystal form 1. MR phases were initially kept up to 8 Å then extended to 3.3 Å resolution by NCS averaging and density modification.

B- 20-fold NCS-averaged electron density of one MglB monomer in crystal form 2. Starting phases were obtained by MR using, as a probe, the density-modified map of crystal form 1. Phases up to 8 Å were initially kept for map calculation and then extended to 2.4 Å resolution by NCS averaging and density modification.

C- Asymmetric unit content of unbound MglB (crystal form 2). 20 independent MglB is arranged as 5 tetramers, colored in shades of red, orange, yellow, blue and green. The 26 C-terminal residues are not visible in any of the monomers.

D- A representative MglB tetramer. The last visible residue in each subunit is labelled C-ter.

E- SEC-MALS analysis shows that MglB is a homogeneous dimer.

F- Plot of $I_{(0)}$ and R_g as a function of frame number for the SEC-SAXS analysis of MglB. Frames 168-187 were selected for data averaging.

G- SEC-SAXS profile of MglB. The insert shows the Guinier plot (q_{max}*Rg=1.19). The radius of gyration R_g is estimated from Guinier analysis.

H- Dimensionless Kratky plot. The shifted peak value (black arrow) and the upwards shape at high qR_g (red arrow) are indicative of an elongated structure with flexible segments.

I- The SAXS profile calculated from the MglB ensemble model with flexible C-terminii calculated with MultiFoXS (blue) has an excellent fit to the the experimental SAXS data (red).

J- Sequence alignment of helices α2 and α3 and C-terminus of MglB from different bacteria species. Conserved positively charged residues are highlighted in yellow. Residues mutated in $MglB^{3M}$ are labelled. Invariant or highly conserved residues in the C-terminus are boxed.

K- Comparison of the MglA/MglB complex and the yeast YPT1-TRAPP complex, showing their common usage of the longin/roadblock dimer to bind their cognate small GTPase.

L- MglB does not stimulate MglA nucleotide exchange. Nucleotide exchange was monitored by fluorescence in the presence (blue) or absence (orange) of MglB. MglA and MglB are at 1µM.

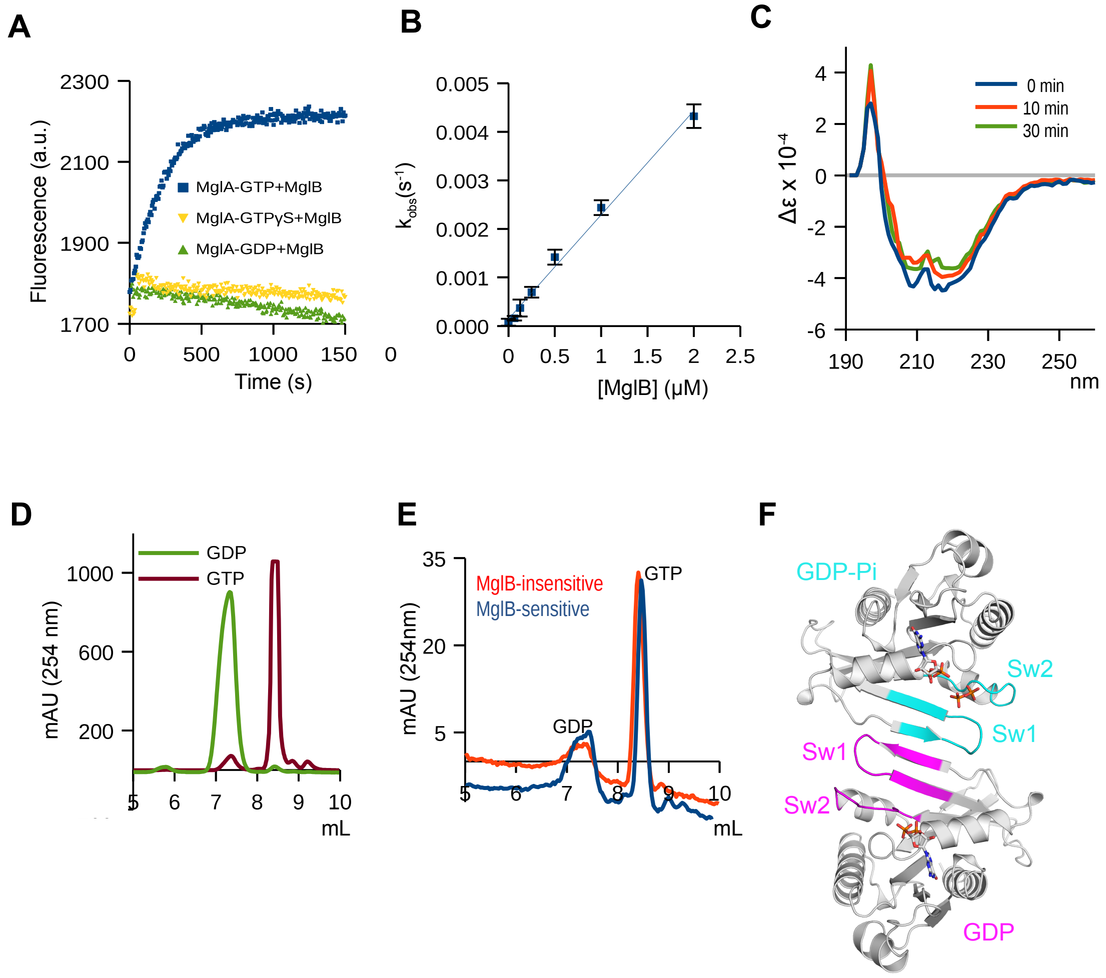

Supplementary Figure 3. **Characterization of the GAP activity of MglB**.

A- Specificity of MglB GAP kinetics measured by fluorescence with the reagentless phosphate-binding protein assay. Production of inorganic phosphate is observed with MglA-GTP (blue), but not with MglA loaded with GTPγS, a non-hydrolyzable GTP analog (red) or with MglA-GDP (green).

B- Determination of k_{cat}/K_m of GTP hydrolysis stimulated by MglB from a linear regression of k_{obs} determined at different MglB concentrations. Individual k_{obs} were determined by mono-exponential fitting.

C- Circular dichroism spectra of MglA-GTP after incubation at 25 °C for 0 (blue), 10 (orange) and 30 (green) minutes. All spectra are representative of a folded protein.

D- Elution volumes of GDP and GTP nucleotides monitored at 254 nm using MonoQ anion exchange chromatography, used as a reference for the experiments shown in **Figure S3E**.

E- MglB-sensitive (incubated on ice, blue) and MglB-insensitive (incubated at 25 °C, red) MglA species contain GTP. MglA-GTP samples were denaturated by addition of methanol and centrifuged to remove precipitated protein before loading on the MonoQ column and analysis of nucleotide content at 254 nm.

F- MglA in the mixed state and MglA-GDP associate as an asymmetric dimer in the crystal through their switch 1 regions. The inactive switch 1 regions are shown in magenta, the active switch 2 in the mixed state is in green, the inactive switch 2 in MglA-GDP is in yellow.

Source data for panel S2B are provided as a Source Data File.

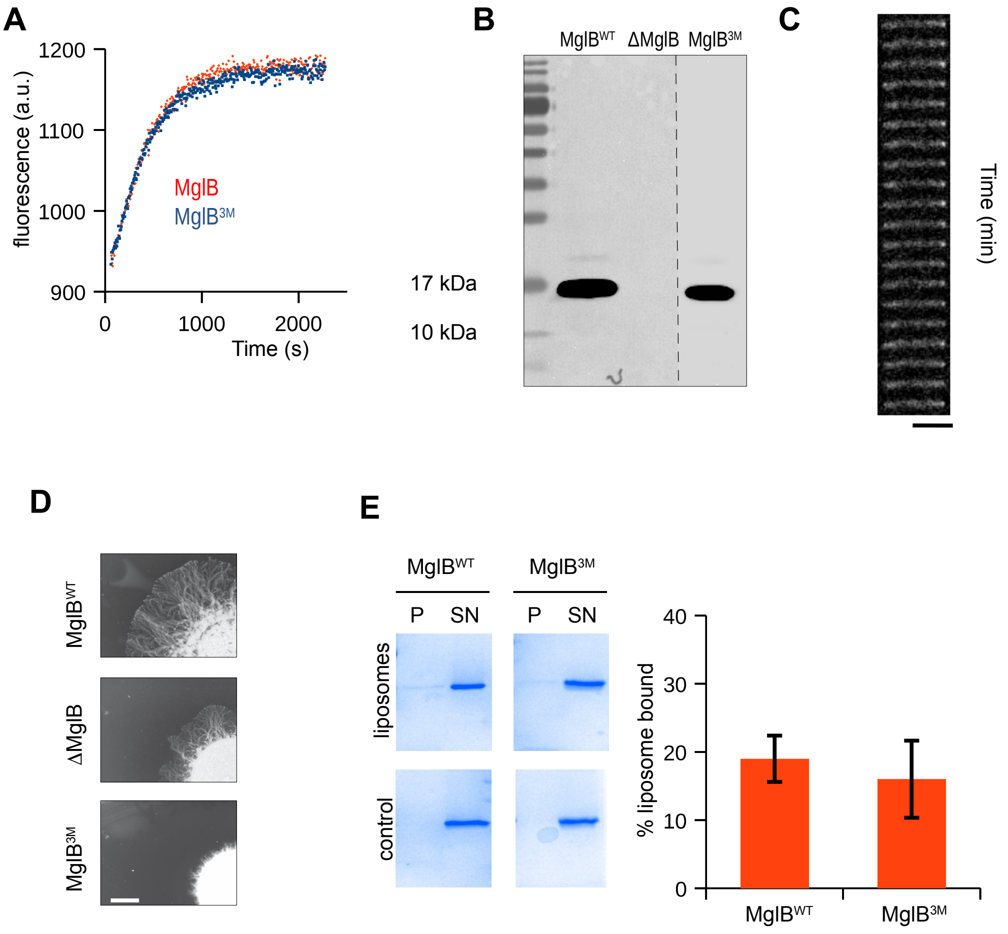

Supplementary Figure 4. **Analysis of the positively charged tract of MglB.**

A- The positively charged tract in MglB is not involved in the GAP activity. GAP kinetics were measured by the reagentless GAP assay as in **Figure 3A**, using an MglB-sensitive MglA-GTP sample. The kinetic profile of wild-type MglB is in red, of MglB^{3M} in blue.

B- Stable expression of MglB^{3M}. Shown is an anti-MglB Western blot. MglB and MglB^{3M} are stably expressed in an *mglB* deletion strain under the control of the *mglB* promoter inserted at the Mx8-phage attachment site. The dotted line shows that the MglB^{3M} blot was spliced closer to the MglB^{WT} and ΔMglB lanes for presentation purposes.

C- MglA-YFP does not switch poles when MglB^{3M} is expressed. Shown is a time lapse of MglA-YFP in an MglB^{3M}-expressing cell. The fluorescence cluster is observed at the same pole during the entire time lapse (representative example of n=35 monitored cells). 1 min time points are shown for a total duration of 20 min. scale bar = 2 µm.

D- Expression of MglB^{3M} leads to profound colony motility defects. Motility on 0.5% Agar is shown for each strain after 48H incubation at 32°C. Scale bar = 1 cm.

E- MglB binds weakly to cardiolipin-containing liposomes and this does not involve the positively charged tract. MglB-liposome interaction was analyzed by co-sedimentation. Pellets (P) containing liposome-bound proteins and supernatants (SN) were analyzed by SDS-PAGE. Controls experiments are carried out without liposomes. Right panel: quantification of the co-sedimentation experiments.

Source data for panels S3B and S3E are provided as a Source Data File.

Supplementary Table 1. Crystallographic data and refinement statistics. Numbers in parentheses refer to the highest resolution shell.

	MglA-GDP	MglA-GTPγS	MglA-GTPγS-MglB	MglA-GDP-Pi/ MglA-GDP (mixed form)	MglB
**Data collection**					
Space group	$P\ 2_1\ 2_1\ 2_1$	I 2 3	$P\ 6_4$	$P\ 2_1\ 2_1\ 2$	$P\ 2_1\ 2_1\ 2_1$
Cell dimensions					
a, b, c (Å)	54.02, 89.2, 118.55	117.35, 117.35, 117.35	135.10, 135.10, 60.38	88.6, 116.65, 49.11	119.78, 139.6, 179.42
α, β, γ (°)	90, 90, 90	90, 90, 90	90, 90, 120	90, 90, 90	90, 90, 90
Resolution (Å)	36.13-1.98 (2.05-1.98)	47.91 - 1.28 (1.32- 1.28)	44.22 - 2.8 (2.9 - 2.8)	48.72 - 2.3 (2.38 - 2.3)	45.41-2.39 (2.48-2.39)
R_{merge}	0.14 (1.28)	0.07 (1.11)	0.08 (1.09)	0.18 (1.48)	0.09 (2.04)
I/σ *I*	13.07 (0.60)	14.01 (1.43)	14.49 (1.90)	8.29 (1.03)	15.36 (1.10)
Completeness (%)	98 (100)	96.2 (63.2)	69.5 (19.9)	74.7 (20.5)	73 (97)
Redundancy	4.4 (4.1)	6.8 (6.8)	6.2 (5.9)	5.3 (4.7)	9.0 (8.9)
**Refinement**					
Resolution (Å)	36.13-1.98 (2.05-1.98)	47.91 - 1.28 (1.32- 1.28)	44.22 - 2.8 (2.9 - 2.8)	48.72 - 2.3 (2.38 - 2.3)	45.41-2.39 (2.48-2.39)
No. Reflections	40036 (3634)	67062 (4392)	10923 (309)	17471 (473)	86633 (1124)
R-work / R-free	0.21 / 0.24	0.16 / 0.18	0.2 / 0.25	0.21 / 0.27	0.26 / 0.29
No. atoms					
Protein	3050	1612	3423	3084	19033
Ligand	58	43	116	138	293
Water	90	331	13	155	0
B-factors ($Å^2$)					
Protein	48.57	19.32	67.10	44.12	70.83
Ligand	31.51	16.19	90.15	55.10	47.18
Water	35.97	36.83	46.85	49.22	0
RMS deviations					
Bond lengths(Å)	0.014	0.006	0.002	0.002	0.012
Bond angles (°)	1.79	0.97	0.62	0.68	1.60

Supplementary Table 2. SAXS data collection and analysis statistics.

X-ray Beamline Source	SWING at SOLEIL Synchrotron, France
Wavelength	1.03 Å
Detector	CCD (AVIEX170170)
Sample-detector distance	1.8 m
Beam geometry	0.4 x 0.1 mm
q range	0.006-0.56 $Å^{-1}$
Sample Injection	HPLC (Bio-SEC3 column)
Temperature	15 °C
Exposure time	2.0 s per frame
**Structural Parameters**	
**From Guinier fit**	
$I_{(0)}$ (cm^{-1})	0.048±6e-05
R_g (Å)	27.61±0.33
qR_g limits	0.63-1.29
**From $P_{(r)}$**	
R_g (Å)	28.5
D_{max} (Å)	96.0
V_P (nm^3)	56.4
M from V_P (kD)	37.6
**Molecular mass determination**	
MglB Monomer Theoretical Value	17.3 kD
SAXS Experiment Estimation	36.5 kD
**Model Evaluation**	
MultiFoxS # (χ^2)	1.22 (e=4)
GASBOR (χ^2)	0.78 (NSD=1.04, n=5)
**SASDBD Accession Code (https://www.sasbdb.org)**	*SASDET9*

Note: # Crystal structure of the MglB dimer with the two C-terminal peptides modeled as ensemble structures.
NSD: Normalized Spatial Discrepancy

Supplementary Table 3. Strains used in this study.

Strain	Construction	Genotype	Source
TM155	DZ2 ΔmglB	ΔmglB	[8]
TM168	TM155 mglB (pSWU19 mglB)	ΔmglB mglB	[8]
TM1199	TM155 mglB^{3M} (pSWU19 mglB^{3M})	ΔmglB mglB^{3M}	This work
TM1264	TM155 mglB mglB-neon green (pSWU19 mglB-neon green)	ΔmglB mglB-neon green	This work
TM1265	TM155 mglB mglB^{3M}-neon green (pSWU19 mglB^{3M}-neon green)	ΔmglB mglB3M-neon green	This work
TM1316	ΔmglB mglA-YFP mglA (pSWU30 mglA) mglB (pSWU19 mglB)	ΔmglB mglA-yfp mglA mglB	This work
TM1319	ΔmglB mglA-YFP mglA (pSWU30 mglA) mglB^{3M} (pSWU19 mglB^{3}M)	ΔmglB mglA-yfp mglA mglB^{3M}	This work

Supplementary Table 4. Plasmids used in this study.

Plasmid	Relevant characteristics	Source or reference
pSUW19	KmR used to integrate genes ectopically at Mx8 att	[9]
pSUW19 MglB	pSWU19 with MglB under MglBpromoter	[8]
pSUW19 mglB3M	pSWU19 with mglB^{3M} under MglBpromoter	This work
pSUW30 mglB-neon green	pSWU30 with mglB-neon greenfusion under MglBpromoter	This work
pSUW19 mglB^{3M}-neon green	pSWU19 with mglB^{3M}-neon green under MglBpromoter	This work

Supplementary Table 5. Primers used in this study.

Name	Sequences (5'_3')
oSL19 (MglB-neon green F1)	ATCCCCGGGTACCGAGCTCGAATTCCACCCCACAGTGCGTCCGGA
oSL56 (MglB-neon green R1)	cgacctcgagggggggcccggtaccCTCGCTGAAGAGGTTGTCGA
oSL57 (MglB-neon green F2)	CGATATCGACAACCTCTTCAGCGAGggtaccgggccccccctcga
oSL58 (MglB-neon green R2)	CAGCTATGACCATGATTACGAATTCtcacttatagagttcatcca
oSL19 (MglB^{3M}-neon green F1)	ATCCCCGGGTACCGAGCTCGAATTCCACCCCACAGTGCGTCCGGA
oSL56 (MglB^{3M}-neon green R1)	cgacctcgagggggggcccggtaccCTCGCTGAAGAGGTTGTCGA
oSL57 (MglB^{3M}-neon green F2)	CGATATCGACAACCTCTTCAGCGAGggtaccgggccccccctcga
oSL58 (MglB^{3M}-neon green R2)	CAGCTATGACCATGATTACGAATTCtcacttatagagttcatcca
oSL19 (MglB^{3M} F1)	ATCCCCGGGTACCGAGCTCGAATTCCACCCCACAGTGCGTCCGGA
oSL23 (MglB^{3M} R1)	CGTACATCACCAGTTGCGTGCCCATGGGCGTATGCGCTCCTCAGC
oSL24 (MglB^{3M} F2)	CGAACGCTGAGGAGCGCATACGCCCATGGGCACGCAACTGGTGAT
oSL25 (MglB^{3M} R2)	CAGCTATGACCATGATTACGAATTCTTACTCGCTGAAGAGGTTGT
MglB-2 (F)	AGC CAT ATG GAG AAT CTT TAT TTT CAG GGC ATG GGC ACG CAA CTG GTG
MglB-2 (R)	GGC TGC GGC CGC TCA CTC GCT GAA GAG GTT GTC
