# Supplementary material for: MglA functions as a three-state GTPase to control movement reversals of Myxococcus xanthus
Source: Nat Commun. 2019 Nov 22;10:5300. doi: 10.1038/s41467-019-13274-3 (PMC6876712; doi:10.1038/s41467-019-13274-3)
Supplement: Supplementary file 2 — Reporting Summary [file 41467_2019_13274_MOESM2_ESM.pdf]

nature research

Corresponding author(s): Jacqueline Cherfils

Last updated by author(s): Sep 10, 2019

Reporting Summary

Nature Research wishes to improve the reproducibility of the work that we publish. This form provides structure for consistency and transparency in reporting. For further information on Nature Research policies, see Authors & Referees and the Editorial Policy Checklist .

Statistics

For all statistical analyses, confirm that the following items are present in the figure legend, table legend, main text, or Methods section.

n/a	Confirmed	
☐	☒	The exact sample size (*n*) for each experimental group/condition, given as a discrete number and unit of measurement
☒	☐	A statement on whether measurements were taken from distinct samples or whether the same sample was measured repeatedly
☒	☐	The statistical test(s) used AND whether they are one- or two-sided<br>*Only common tests should be described solely by name; describe more complex techniques in the Methods section.*
☒	☐	A description of all covariates tested
☒	☐	A description of any assumptions or corrections, such as tests of normality and adjustment for multiple comparisons
☒	☐	A full description of the statistical parameters including central tendency (e.g. means) or other basic estimates (e.g. regression coefficient) AND variation (e.g. standard deviation) or associated estimates of uncertainty (e.g. confidence intervals)
☒	☐	For null hypothesis testing, the test statistic (e.g. *F*, *t*, *r*) with confidence intervals, effect sizes, degrees of freedom and *P* value noted<br>*Give P values as exact values whenever suitable.*
☒	☐	For Bayesian analysis, information on the choice of priors and Markov chain Monte Carlo settings
☒	☐	For hierarchical and complex designs, identification of the appropriate level for tests and full reporting of outcomes
☒	☐	Estimates of effect sizes (e.g. Cohen's *d*, Pearson's *r*), indicating how they were calculated

Our web collection on statistics for biologists contains articles on many of the points above.

Software and code

Policy information about availability of computer code

Data collection	Crystallographic data were collected on synchrotrons SOLEIL and ESRF, beamlines PX1, PX2 and ID30b
Data analysis	Data analysis was performed under Image J using open source plugins such as MicrobeJ. Graphs were plotted in Microsoft Excel v16.9 or LibreOffice 6.0; kinetics were analyzed using GraphPad Prism v7.0. Diffraction data were processed with autoPROC or xdsme; Molecular replacement was performed with Phaser from the Phenix suite and refinements were performed using phenix.refine and Coot; superpositions of crystallographic structures were performed with Pymol v2.2.0

For manuscripts utilizing custom algorithms or software that are central to the research but not yet described in published literature, software must be made available to editors/reviewers. We strongly encourage code deposition in a community repository (e.g. GitHub). See the Nature Research guidelines for submitting code & software for further information.

Data

Policy information about availability of data

All manuscripts must include a data availability statement. This statement should provide the following information, where applicable:

- Accession codes, unique identifiers, or web links for publicly available datasets
- A list of figures that have associated raw data
- A description of any restrictions on data availability

The authors declare that all data supporting the findings of this study are available within the paper and its supplementary information files.

Field-specific reporting

Please select the one below that is the best fit for your research. If you are not sure, read the appropriate sections before making your selection.

Life sciences study design

All studies must disclose on these points even when the disclosure is negative.

Sample size	The chosen sample sizes produced suitable standard deviations.
Data exclusions	No data were excluded
Replication	All attemps at biochemical and motility replication were successful
Randomization	Not relevant
Blinding	Not relevant

Reporting for specific materials, systems and methods

We require information from authors about some types of materials, experimental systems and methods used in many studies. Here, indicate whether each material, system or method listed is relevant to your study. If you are not sure if a list item applies to your research, read the appropriate section before selecting a response.

Materials & experimental systems

n/a	Involved in the study	
☐	☒	Antibodies
☒	☐	Eukaryotic cell lines
☒	☐	Palaeontology
☒	☐	Animals and other organisms
☒	☐	Human research participants
☒	☐	Clinical data

Methods

n/a	Involved in the study	
☒	☐	ChIP-seq
☒	☐	Flow cytometry
☒	☐	MRI-based neuroimaging

Antibodies

Antibodies used	MglB antibodies were used as described in Zhang et al. Plos Biology 2010 doi: 10.1371/journal.pbio.1000430
Validation	MglB antibody validation was provided in Zhang et al. Plos Biology 2010 doi: 10.1371/journal.pbio.1000430
